# Anti-Inflammatory Activity of Extracts and Pure Compounds Derived from Plants via Modulation of Signaling Pathways, Especially PI3K/AKT in Macrophages

**DOI:** 10.3390/ijms21249605

**Published:** 2020-12-16

**Authors:** Anna Merecz-Sadowska, Przemysław Sitarek, Tomasz Śliwiński, Radosław Zajdel

**Affiliations:** 1 Department of Computer Science in Economics, University of Lodz, 90-214 Lodz, Poland; 2 Department of Biology and Pharmaceutical Botany, Medical University of Lodz, 90-151 Lodz, Poland; przemyslaw.sitarek@umed.lodz.pl; 3 Laboratory of Medical Genetics, Faculty of Biology and Environmental Protection, University of Lodz, 90-236 Lodz, Poland; 4 Department of Medical Informatics and Statistics, Medical University of Lodz, 90-645 Lodz, Poland; radoslaw.zajdel@uni.lodz.pl

**Keywords:** medicinal plants with economic value, plant-derived compounds, inflammation, macrophages, signaling pathways, PI3K/AKT pathway

## Abstract

The plant kingdom is a source of important therapeutic agents. Therefore, in this review, we focus on natural compounds that exhibit efficient anti-inflammatory activity via modulation signaling transduction pathways in macrophage cells. Both extracts and pure chemicals from different species and parts of plants such as leaves, roots, flowers, barks, rhizomes, and seeds rich in secondary metabolites from various groups such as terpenes or polyphenols were included. Selected extracts and phytochemicals control macrophages biology via modulation signaling molecules including NF-κB, MAPKs, AP-1, STAT1, STAT6, IRF-4, IRF-5, PPARγ, KLF4 and especially PI3K/AKT. Macrophages are important immune effector cells that take part in antigen presentation, phagocytosis, and immunomodulation. The M1 and M2 phenotypes are related to the production of pro- and anti-inflammatory agents, respectively. The successful resolution of inflammation mediated by M2, or failed resolution mediated by M1, may lead to tissue repair or chronic inflammation. Chronic inflammation is strictly related to several disorders. Thus, compounds of plant origin targeting inflammatory response may constitute promising therapeutic strategies.

## 1. Introduction

Plant-derived products have become increasingly popular in recent years as alternatives to traditional pharmaceuticals [1,2]. Their regulative effects are a consequence of naturally occurring compounds that belong to secondary plant metabolites. That group includes phenolics, alkaloids, saponins, terpenes, lipids and carbohydrates [3]. Phytochemicals isolated from plants are characterized by numerous biological activities, including an anti-inflammatory property [4]. Studies have suggested that the impact of naturally occurring compounds on inflammation processes may be related to their regulatory effect on macrophages biology [5]. 

Macrophages are important immune cells that are located throughout the tissues of the body and are developed from monocytes [6]. They are crucial components of an inflammatory response and take part in antigen presentation, phagocytosis, immunomodulation and tissue repair [7]. Macrophages, depending on their environment, may be polarized into classically activated M1 with proinflammatory properties and alternatively activated M2 with anti-inflammatory properties. Therefore, the modulation of their phenotype may impact initiation, development and resolution of inflammatory processes [8]. Inflammation is strictly related to numerous disorders, including diabetes, cardiovascular diseases, chronic respiratory diseases, arthritis, cancer and obesity [9]. Thus, the macrophages phenotype may influence the progress of these diseases. Furthermore, molecules that target their differentiation may hold an important purpose for novel therapeutic strategies.

The important factors that influence macrophage polarization include specific microbial products and cytokines secreted during inflammation [10]. Moreover, numerous additional stimuli may mediate macrophage differentiation. Regulation has been shown to be apparent on the signaling pathways. One of those pathways, the phosphoinositide 3-kinase/protein kinase B (PI3K/AKT) cascade, is triggered in immune cells by the binding of a lipopolysaccharide (LPS), an important component of bacteria, into its specific receptor, named Toll-like receptor 4 (TLR4), that recognizes pathogen-associated molecular patterns (PAMPs) [11]. Protein kinase B (AKT or PKB) and a mammalian target of rapamycin complex 1 (mTORC1) are downstream targets [12]. PI3K activity is related to M2 phenotype [13]. The action of AKT depends on their isoform. Deletion of AKT1 promotes M1 macrophages, whereas ablation of AKT2 gives rise to M2 macrophages phenotype [14]. Induction of mTORC1 signaling promotes M2 macrophages polarization [15], but it is also related to inhibition of autophagy, a potent anti-inflammatory mechanism [16]. In turn, PI3K signal transduction may be modulated by several plant extracts as well as pure compounds. Molecules that aim at macrophage biology may be an important immune target and constitute a crucial approach in therapeutic strategies against inflammatory disorders.

Therefore, in this study, we present a summary of research progress on anti-inflammatory activity of the extracts and pure compounds derived from plants with a regulatory effect on macrophages via modulation of signaling pathways, especially PI3K/AKT.

## 2. Medicinal Plants with Economic Value

Plants species that are beneficial for humans are named economic plants. They include plants utilized as nutrients and therapeutic agents [17]. Plants that contain chemicals that can be employed for therapeutic purposes are named medicinal plants. Herbal medicine is the use of medicinal plants for prevention and/or treatment of numerous diseases. Traditional herbal medicine has a long history that extends as far back as at least 60,000 years. Chinese medicine, Ayurveda, Kampo, traditional Korean medicine and Unani use natural products and are a valuable collection of human knowledge. The defense properties of medical plants include a wide and diverse variety of active compounds [18,19].

Phytochemical constituents are divided into two categories: primary and secondary metabolites. The first group of compounds includes an important metabolite for plants life functions, whereas the second one mainly plays a supportive role in interaction with the external environment [20]. Secondary metabolites, depending on their chemical structures, are classified into the following groups: phenolics, alkaloids, saponins, terpenes, lipids and carbohydrates. Groups may be then divided into classes and subclasses. Phenolics are comprised of simple phenolics, tannins, coumarins, flavonoids, chromones and xanthones, stilbenes and lignans. Terpenes are classified into hemiterpenes, monoterpenes, sesquiterpenes, diterpenes, sesterterpenes and triterpenes. The lipid group includes fixed oils, waxes and essential oils. Carbohydrates are divided into monosaccharides, disaccharides, oligosaccharides and polysaccharides [21].

Medical plants are an important pool for discovery of new active compounds with a therapeutic potential [22]. However, important challenges implicated in the use of the plants are their accessibility and quality of materials, determining chemical composition, identification of structures of bioactive molecules and characterization of molecular mechanisms of action. Despite those difficulties, the interest in phytochemicals is still rising. Plant extracts or single isolated compounds are reported to possess numerous biological properties; in particular, natural chemicals are gaining special attention as cell signaling pathway modifying agents [23]. The therapeutic agents originated from plants are as follows: aspirin, codeine, papaverine, colchicine, digoxin and digitoxin, cannabidiol, tetrahydrocannabinol, vinblastine and vincristine, artemisinin, galantamine, apomorphine hydrochloride, tiotropium bromide, paclitaxel, camptothecin and allicin [24]. Plant-based products possess numerous contributing properties, such as antimicrobial [25], antiobesity [26], antioxidant [27], anti-inflammatory [28] or anticancer [29] properties. Moreover, the chemical diversity of natural compounds allows creation of new molecules of plant origin, which also offer several bioactivities [30]. The use of natural products is an important strategy of bioeconomy that facilitate economic upgradation, especially in nations with rich plant resources [30].

The alternative approach to obtain favorable molecules is a semisynthesis, where most of the molecular skeleton serves as precursor for derivatives. Examples of the semisynthesis strategy are: a terpene named artemisinin, isolated from *Artemisia annua* with antimalarian potential [31], and an alkaloid named cyclopamine, isolated from *Veratrum californicum*, an antagonist of the Hedgehog signaling pathway that blocks the G-protein-coupled receptor called Smoothened and possesses anticancer potential. Moreover, an alkaloid named neothiobinupharidine, isolated from *Nuphar luteum*, that exhibit antimetastatic activity, terpenoid named hyperforin, isolated from *Hypericum perforatum*, with antidepressant activity and the ester of diterpenoid named ingenol, isolated from *Euphorbia ingens,* with a potential function against actinic keratosis, may be obtained through transform-based strategies. That approach enables creation of derivatives via replacement of the structural subunit of favorable ones [32]. 

The annual worldwide market for natural products is growing. It is estimated to be USD 1 trillion for medical supplies, functional foods, etc. [33], whereas medicinal plants products approached USD 100 billion per annum [34]. The global export value of 50,000 to 70,000 plant species with suspected medicinal properties was estimated at USD 2.2 billion in 2012 [33]. Medical plants exist in numerous different forms, including syrup, ointments, capsules and tablets. [35]. 

## 3. Macrophage Characterization and Activation

Macrophages display phagocytic and immunomodulatory properties, and they contribute to antigens’ presentation and regulation of tissue homeostasis. They are localized in different organs and tissues such as bone, lung, central nervous system, connective tissue, liver, peritoneum and adipose tissue, and are named osteoclasts, alveolar macrophages, microglial cells, histiocyte, Kupffer cells, peritoneal macrophages and adipose tissue macrophages, respectively. The origins of those cells are myeloid progenitor cells, which are located in the bone marrow that differentiate to peripheral blood mononuclear cells that migrate into the tissue after inflammatory stimuli and then developed into macrophages [36]. Macrophage production regulates colony stimulating factor 1 (M-CSF-1). M-CSF-1 administration is induced by phosphorylation of extracellular signal-regulated protein kinase (ERK) 1/2, a member of the mitogen-activated protein kinase (MAPK) family [37].

**Figure 1:**
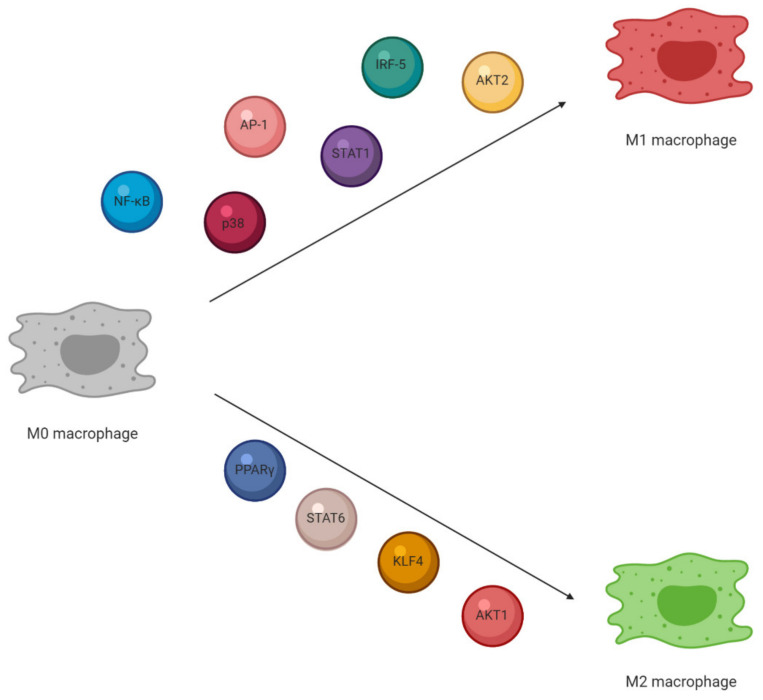
Selected regulators of macrophage polarization (created by BioRender.com).

The roles of M1 macrophages are the clearance of pathogens and microbicidal activity; M2 macrophages clear other cellular materials and promote tissue repair activity; M2a macrophages regulate inflammatory response and possess anti-inflammatory activity [10]. Those three groups of cells are activated by different stimuli (Figure 1). Classically activated M1 macrophages with proinflammatory properties are induced by microbial products including lipopolysaccharide (LPS) as well as interferon gamma (IFN-γ) secreted by T helper 1 (Th-1) lymphocytes during adaptive immune response processes and IFN-γ secreted by natural killer (NK) cells during innate immune response processes. Moreover, their population rises also in response to tumor necrosis factor (TNF-α) released by antigen-presenting cells (APCs). Alternatively, activated M2 macrophages with anti-inflammatory properties rise in response to IL-4, which can be produced by mast cells and basophils during innate immune response, IL-4 and IL-13 produced by Th-2 lymphocytes during adaptive immune response processes. Macrophages with anti-inflammatory activity named regulatory macrophages or M2a are activated by numerous stimuli such as immune complexes, G-protein coupled receptor (GPCR) ligands, prostaglandins, glucocorticoids, apoptotic cells, transforming growth factor beta (TGF-β) or IL-10 release by regulatory T cell. 

As a result of activation, macrophages produce numerous pro- and anti-inflammatory cytokines. M1 macrophages produce pro-inflammatory agents, such as IL1-β, IL-6, IL-12, IL-23, TNF-α, inducible nitric oxide synthase (iNOS) and cyclooxygenase 2 (COX-2). Additionally, their initiation promotes T lymphocytes antigens presentation and activation of adaptive immune response. M2 macrophages secrete anti-inflammatory factors such as TGF-β and insulin-like growth factor 1 (IGF-1). That action inhibits inflammation and promotes tissue remodeling and repair. Induction of M2a macrophages propagate modulation of inflammatory reaction, but macrophages of that activated subgroup secrete both pro- and anti-inflammatory agents, such as IL-1β, IL-10, TNF-α and TGF-β [36,38]. 

M1 macrophages utilize iNOS to obtain 1-citruline and nitric oxide (NO) from 1-arginine. NO is essential for their cytotoxic activity [39,40]. Mitochondria and nucleus are important targets of NO, which can interact directly with nuclear DNA. Moreover, NO modulates mitochondrial membrane permeability and transfer chain. As a consequence, necrosis or apoptosis of invader cells is triggered [41]. In turn, M2 macrophages utilize arginase, which hydrolyzes arginine to ornithine and urea. Limited arginine reduces NO synthesis, whereas ornithine plays a role in downstream pathways of polyamine and proline syntheses which contribute to cell proliferation and tissue repair [40]. 

Apart from specific microbial products and cytokine secreted in inflammation, other agents are also crucial in macrophage differentiation. Important M1 macrophage stimuli include a nuclear factor of kappa light polypeptide gene enhancer (NF-κB), MAPKs, activator protein 1 (AP-1), signal transducer and activator of transcription 1 (STAT1), interferon regulatory factors (IRF) 5 and serine/threonine-protein kinase (AKT) 2, whereas M2 macrophages stimuli are as follows: STAT6, IRF4, peroxisome proliferator-activated receptor (PPAR) γ, Krüppel-like factor (KLF) 4 and AKT1 [8,42,43] (Figure 1).

## 4. Plants and Their Active Compounds as Modulators of Macrophage Polarization

The interaction between phagocyte and microbe is a host defense mechanism, but excessive reactivity may impair tissue, followed by a development of autoimmune problems, allergies, rheumatoid disorders and even cancer, among others. Plants are the source of molecules capable to modulate immune response via different mechanisms of action, including a direct impact on immune cells. The immunomodulating potential of phytochemicals may be used to treat immune-related diseases. Additionally, clinical application of phytochemicals as modulators of host immunity against cancer cells may be a prospective approach in anticancer therapy [44,45]. 

Several groups of secondary plant metabolites have an impact on macrophages’ biology, including phenolic compounds, terpenes and polysaccharides. The data indicate that M1 human macrophages incubated with flavonoids such as quercetin, naringenin and naringin exhibit downregulation of glycolytic activity [46]. RAW 264.7 cells stimulated by lipopolysaccharide (LPS) exhibit excessive levels of proinflammatory mediators. Pretreatment with terpenes, including d-limonene, linalool, terpinolene, α-terpineol, α-phellandrene and γ-terpinene, lead to their significant reduction [47]. The overall activity of plant polysaccharides enhances macrophage immune response via reactive oxygen species (ROS) production and excessive secretion of cytokines and chemokines [48].

The data indicated that plant-derived active compounds are also able to modulate macrophage polarization [49,50]. The examples of naturally occurring compounds that are able to regulate M1 polarization are: diosgenin, a steroidal sapogenin isolated from the *Tritulus terrestris*; polysaccharides extracted from the *Citrus grandis*; lupeol, a pentacyclic triterpene derived from the *Tamarindus indica* and *Sebastiania adenophora*. Natural regulators of M2 polarization are as follows: celastrol, a triterpene isolated from the *Tripterygium wilfordii*; luteolin, a flavone derived from several plant species; curcumin, a compound extracted from the *Curcuma longa*; isoliquiritigenin, a flavonoid found in licorice and punicalagin; ellagitannin, a hydrolysable tannin isolated from pomegranate. Moreover, salidroside, a glycoside isolated from the *Rhodiola rosean* and *Trichosanthes kirilowii*, lectin and 1,3,6,7-tetrahydroxy-8-prenylxanthone extracted from *Garcinia mangostana* regulate both M1/M2 polarization [5].

Responsiveness of the cells to the external stimuli is followed by regulation of gene expression [51]. Data indicate that plants and their active compounds may be modulators of the transcription process. RAW267.4 murine cells stimulated by hydrogen peroxide upregulate the transcription of several proinflammatory mediators such as COX2, IL1β, NFκB, NOS and TNFα, while PPARγ is inhibited. Tested macrophages after treatment with *Arctium lappa*, *Camellia sinensis*, *Echinacea angustifolia*, *Eleutherococcus senticosus*, *Panax ginseng* and *Vaccinium myrtillus* extracts exhibited modulation of the release of the abovementioned mediators and in vitro anti-inflammatory activity [52]. Simultaneously, PPARγ-mediated gene expression is upregulated and NO production is suppressed by *Maerua subcordata* extract in LPS stimulated RAW264.7 murine macrophages [53]. The cells treated with *Archidendron lucidum* extract exhibit downregulation of expression iNOS, COX-2, TNF-α, IL-1β and IL-6 [54]. The other study indicate that cottonseed extract, an important source of polyphenols, stimulate an anti-inflammatory tristrataprolin family gene expression in mouse RAW264.7 macrophages [55]. Alveolar macrophages of pigs fed with capsicum, garlic botanical or turmeric oleoresin and infected with porcine reproductive and respiratory syndrome virus exhibit altering genes expression, especially in genes responsible for immunity [56]. Moreover, signaling pathways are able to control processes of translation in macrophages that regulate macrophages biology [57]. Gene expression may be modulated by interacting with multiple signaling pathways [58,59].

### 4.1. M1 Macrophages Stimuli and Plant-Based Modulators

The important M1 macrophages stimuli include NF-κB, MAPKs, AP-1, STAT1, IFN-5 and AKT2. The factors that were modulated by natural compounds were taken into consideration.

**Figure 2:**
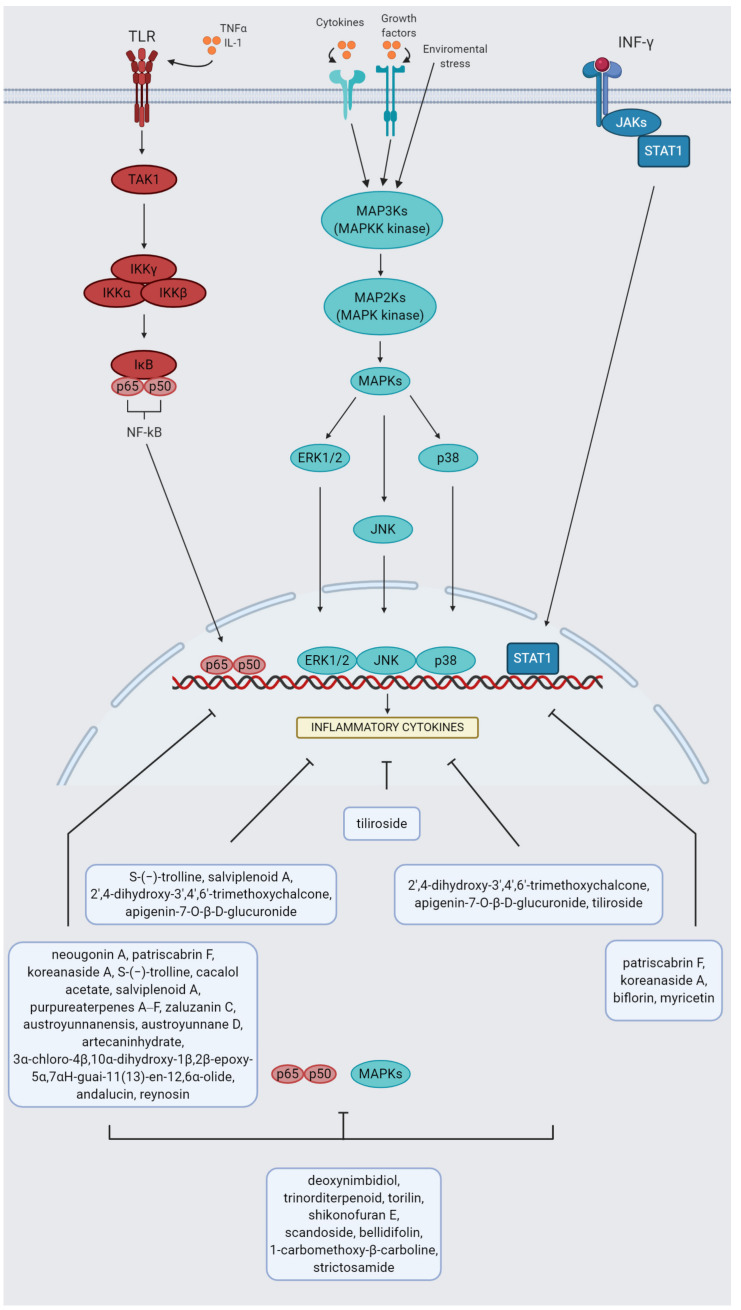
NF-κB, MAPKs, JAK/STAT1 pathways and plant-derived inhibitors (created by BioRender.com).

TLR4 activation induce one of the adaptor proteins, named myeloid differentiation primary response gene 88 (MyD88). That triggers the NF-kB canonical pathway [60]. The crucial proinflammatory NF-kB protein members are heterodimers p65 and p50. The precursor of p50 is named p105 [61]. In a steady state, NF-κB dimers are located in the cytoplasm and bound to inhibitor protein IκB. After activation, IκB kinase (IKK) complex composed of IKKα and IKKβ, and IKKγ (NEMO) subunit with regulatory function phosphorylate IκB protein that is followed by their proteasomal degradation. Then, NF-κB dimers are released and translocated into nucleus [62]. NF-kB induction promotes macrophage relocation at the site of infection and proinflammatory gene induction. NF-κB target genes are as follows: IL-1, IL-2, IL-6, IL-8, IL-12 and TNF-α [51,63]. One of its downstream protein is iNOS. It was shown that inhibition of IKKβ in tumor-associated macrophages switched the macrophage phenotype from M2 to M1, that was related to increased IL-12 and iNOS release [64]. NF-kB signaling cascade is a molecular target for numerous plant extracts or single-derived compounds from species belonging to different families. The examples of natural downregulators of that pathway in macrophages with anti-inflammatory potential are as follow: *Moringa oleifera* [65], *Piper cubeba* [66], *Terminalia argentea* [67], *Vitis vinifera* [68], *Petiveria alliacea* [69], *Panax ginseng* [70], *Dipsacus asperoides* [71], *Humulus lupulus* [72] or *Astilbe chinensis* [73] extracts. The studies were conducted on LPS-induced RAW 264.7 macrophages. Recently, it has been indicated that numerous plant-derived compounds of different groups may inhibit NF-κB signaling [74]. Examples include: neougonin A, a flavonoid obtained from *Helminthostachys zeylanica*; patriscabrin F, an iridoid obtained from *Patrinia scabra* [75]; koreanaside A, a lignan isolated from *Forsythia koreana* [76]; S-(−)-trolline, an alkaloid from *Tetrastigma hemsleyanum* [77]; cacalol acetate, a sesquiterpene from *Psacalium decompositum* [78]; salviplenoid A, a sesquiterpene from *Salvia plebeian* [79]; purpureaterpenes A‒F, sesquiterpenes isolated from the *Echinacea purpurea* [80]; zaluzanin C, a sesquiterpene lactone from *Ainsliaea acerifolia* [81]; austroyunnanensis, austroyunnane D, artecaninhydrate, 3α-chloro-4β,10α-dihydroxy-1β,2β-epoxy- 5α,7αH-guai-11(13)-en-12,6α-olide, andalucin and reynosin, sesquiterpene lactones obtained from *Artemisia austroyunnanensis* [82] (Figure 2).

MAPKs is a serine/threonine protein kinase family, among others engaged into regulation of macrophage activities. MAPKs include ERK1/2, c-Jun N-terminal kinase (JNK) and p38 kinases that are activated via numerous stimuli, including environmental stress, growth factors and cytokines. Activation of MAPKs occurs by phosphorylation via MAP kinase kinase (MKK), which is phosphorylated via MAPK kinase kinase (MKKK). Each of the subclasses is activated in a distinct pathway and further phosphorylate specific substrates [83]. ERK1/2 participates in macrophages development [37]. ERK1/2 is suppressed by *Dipsacus asperoides* extract [71], as well as S-(−)-trolline, an alkaloid isolated from *Tetrastigma hemsleyanum* [77], salviplenoid A, a sesquiterpene from *Salvia plebeian* [79], 2′,4-dihydroxy-3′,4′,6′-trimethoxychalcone, a chalcone from *Chromolaena odorata* [84] and apigenin-7-*O*-β-d-glucuronide, a flavonoid derivative from *Juglans sigillata* [85]. JNKs contain JNK1, JNK2 and JNK3 proteins. JNK is inhibited by tiliroside, a glycosidic flavonoid from *Agrimonia pilosa* [86]. Another MAPK family, named p38, includes four members: p38α, p38β, p38γ and p38δ [87]. P38 is involved in the production of proinflammatory agents, such as TNF-α and COX-2 [88]. Activation of p38 promotes M2 into M1 tumor-associated macrophages repolarization, whereas inhibition impairs M2 polarization [89]. P38 level is downregulated in RAW264.7 macrophages by *Panax ginseng* extract [70] as well as 2′,4-dihydroxy-3′,4′,6′-trimethoxychalcone, a chalcone from *Chromolaena odorata* [84], apigenin-7-*O*-β-d-glucuronide, a flavonoid derivative from *Juglans sigillata* [85] and tiliroside, a glycosidic flavonoid from *Agrimonia pilosa* [86]. Moreover, ERK, JNK and p38 are a molecular targets in in vitro study conducted on LPS-stimulated RAW264.7 macrophages treated with peanut sprout [90] extract (Figure 2).

Downstream-targeted MAPKs include AP-1 family protein [91]. AP-1 grouped transcription factors that, after translocation to the nucleus, induce gene expression of proinflammatory cytokines. Moreover, the study indicates that inhibition of NOS is implicated in prevention of their translocation [92]. AP-1 is a mediator of M1 macrophage polarization [93]. That transcription factor is a target for *Persicaria chinensis* and *Hovenia dulcis* extracts in a study conducted on RAW264.7 cells [94,95]. AP-1 protein is also downregulated in LPS-activated RAW 264.7 macrophages by patriscabrin F, an iridoid obtained from *Patrinia scabra* [75], koreanaside A, a lignan isolated from the *Forsythia koreana* [76] and torilin, a sesquiterpene from *Torilis japonica* [96].

**Figure 3:**
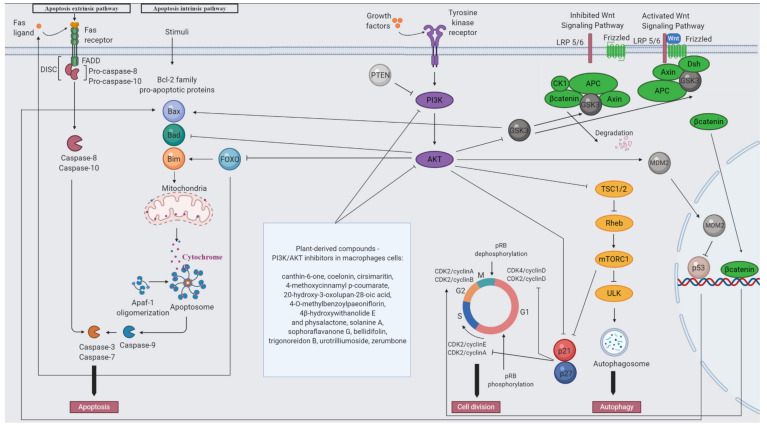
PI3K/AKT signaling pathway with plant-based inhibitors (created by BioRender.com).

Both NF-κB and MAPK activation were significantly suppressed by *Passiflora foetida* [97], *Heracleum moellendorffii* [98], *Vaccinium oldhamii* [99], *Dendropanax morbifera* [100], *Zingiber zerumbet* [101], *Apios americana* [102]; *Rosa rugosa* [103], *Phyllanthus amarus* [104], *Punica granatum* [105], *Castanea seguinii* [106], *Melandrii herba* [107] and *Acmella oleracea* [108] extracts in macrophages. The NF-κB pathway and additionally ERK1/2 and JNK were inhibited by *Aster incisus* [109] and *Fructus sophorae* [110] extracts; ERK1/2 and p38 were attenuated by *Hibiscus syriacus* [111] and *Rodgersia podophylla* [112] extracts; JNK and p38 were decreased by *Populus deltoides* [113] and *Xanthii fructus* [114] extracts in macrophage cell lines. Furthermore, deoxynimbidiol and trinorditerpenoid, diterpenoids from *Celastrus orbiculatus* [115], torilin, a sesquiterpene obtained from *Torilis japonica* [96], shikonofuran E, a phenolic compound isolated from *Onosma paniculatum* [116], scandoside, an iridoid from *Hedyotis diffusa* [117], bellidifolin, a xanthone from *Swertia chirayita* [118], 1-carbomethoxy-β-carboline, an alkaloid from *Portulaca oleracea* [119] and strictosamide, an alkaloid from *Nauclea officinalis* [120] inhibited both NF-κB and MAPK signaling (Figure 3). 

The Janus kinase (JAK)-signal transducer and activator of transcription (STAT) pathway (JAK/STAT) is activated by cytokines. Data indicate that IFN-γ is one of the most critical endogenous macrophage-activating agents. Interferon gamma (IFN-γ) and TLR4 receptor bounding induce JAK. Activated JAK then phosphorylates STATSs, leading to creation of dimers as well as a cytokine receptor and enabling STATs docking. Dimerized STATs translocate into the nucleus and their role of transcription factors allows to regulation of the expression of, among others, cytokine-related genes. JAK families involve four members (JAK1, JAK2, JAK3, TYK2), whereas STATs involve seven (STAT1, 2, 3, 4, 5a, 5b, 6) [121]. STAT1 initiates transcription of genes typical for M1 macrophages and results in a release of proinflammatory cytokines [122]. Activation of STAT1 was inhibited for the *Pueraria montana* [123] extract in mouse peritoneal macrophages and *Chaenomeles sinensis* [124] in LPS-stimulated RAW264.7 cells. Additionally, RAW 264.7 macrophages stimulated by LPS treated with patriscabrin F, an iridoid isolated from the *Patrinia scabra* [75], koreanaside A, a lignan obtained from the *Forsythia koreana* [76], biflorin, a chromone C-glucosides from *Syzygium aromaticum* [125] and myricetin, a flavonoid from *Diospyros lotus* [126] also exhibit suppression of STAT1 protein level (Figure 2).

Interferon-regulatory factors (IRFs) play a key role in regulation of the IFN system. They are essential for control of gene expression related to IFN type I production [127]. IRFs contain nine members, and IRF-5, among others, is involved in M1 polarization. IRF-5 is induced in response to numerous proinflammatory stimuli and mediate the release of inflammatory cytokines, including TNF-α, IL-6 and IL-12. In monocyte-derived macrophages, stimulation of IRF-5 expression is induced by granulocyte-macrophage colony-stimulating factor (GM-CSF) [128]. Mangiferin, a polyphenol present in *Mangifera indica*, has an inhibitory effect on IRF-5 expression in the THP-1 human monocyte cell line and suppresses macrophage classical activation [129], whereas baicalin, a flavone glycoside presented in *Scutellaria baicalensis,* has a similar effect on murine peritoneal macrophages and modulates polarization of macrophages into the M2 phenotype [130]. 

### 4.2. M2 Macrophages Stimuli and Plant-Based Modulators

The important M2 macrophages stimuli include STAT6, IRF-4, PPARγ, KLF4 and AKT1. The factors that were modulated by natural compounds were taken into consideration.

STAT6 signaling is activated by IL-4 and IL-13, and it promotes M2 polarization [131]. STAT6 induction is related to decreased responsiveness to LPS after IL-4 stimuli. As a consequence, macrophages exhibit reduced inflammasome activation and IL-1β production followed by pyroptosis [132]. Increased STAT6 expression is related to anti-inflammatory properties of *Mahonia oiwakensis* extract as well as their major components, berberine and palmatine, the alkaloid compounds in activated macrophage RAW264.7 cells and mouse splenic macrophages. In turn, downregulation of STAT6 were observed in mouse alveolar macrophage cells after exposure to 18β-glycyrrhetinic acid, an pentacyclic triterpenoid and important component of *Glycyrrhiza glabra* that exert immunomodulatory effect [133].

IRF-4 play a role in M2 macrophage polarization. IRF-4 is activated via a downstream target of PI3K-dependent signaling, named 3-phosphoinositidedependent protein kinase 1 (PDPK1). IRF-4 mediates IL-4 and IL-5 production. Their expression in monocyte-derived macrophages is induced by M-CSF. IRF-4 and IRF-5 compete for binding to MyD88 and decide about final macrophages’ phenotypes. Upregulation of IRF-4 expression and polarization to an M2 phenotype was observed in murine peritoneal macrophages treated with baicalin, a flavone originally obtained from *Scutellaria baicalensis* [130].

PPARs are transcription factors, members of nuclear hormone receptors superfamily, following three subtypes: PPARα, PPARγ and PPARβ/δ. PPARγ is induced by some eicosanoids. Activation causes insulin sensitization and elevates glucose metabolism [134]. PPAR controls the inflammatory response by negatively interfering with proinflammatory signaling pathways, such as AP-1 or NF-κB, in activated M1 macrophages [42]. PPARγ directly interacts with p65/p50 in macrophages and inhibits production of IL-12 stimulated by LPS [135]. Data indicate that inactivation of NF-κB is related to p65 proteolytic degradation. PPARγ inhibits AP-1 binding activity into DNA and, as a consequence, suppresses COX-2 protein expression [136]. Moreover, PARP promotes the expression of crucial antioxidant enzymes, including superoxide dismutase, catalase and heme oxygenase-1, decreasing the level of ROS [136]. Overproduction of ROS may result in cell injury and contribute to inflammation [137]. The induction of PPARγ gene expression was mediated by *Maerua subcordata* extract in LPS-induced RAW264.7 macrophages [53]. Additionally, a similar effect was observed in those cells after administration of smiglaside A, a glycoside isolated from the *Smilax riparia* [138], and chrysophanol, an anthraquinone derivative obtained from *Rheum palmatum* [139].

KLFs are a family of transcription factors that have a characteristic zinc-finger domain. The family contains 17 members [140]; some of the members are responsible for macrophage polarization regulation. KLF4 promotes macrophage polarization from the M1 state to the M2 state [141]. KLF4 crosstalks with STAT6 to induce M2 polarization and, with coactivators essential for NF-κB signaling induction, to inhibit M1 polarization. KLF4 knockdown macrophages exhibit increased proinflammatory gene expression [142]. KLF4 is regulated by a microRNA named miR-375 that, when overexpressed, leads to a suppression of KLF4 [143]. It was detected that KLF4 is activated by tanshinone IIA, an abietane diterpenoid presented in *Salvia miltiorrhiza,* thereby inhibiting miR-375 in the RAW 264.7 macrophage [144].

## 5. The PI3K/AKT Signaling Pathway

### 5.1. PI3K/AKT Signaling Modulates Macrophage Activation

The PI3K pathway (Figure 3) plays a pivotal role in the physiology of macrophages. It activates numerous proteins by generation of phosphatidylinositol-3,4,5-trisphosphate (PI(3,4,5)P3) via phosphorylation of phosphatidylinositol-4,5-bisphosphate (PI(4,5)P2) at the 3′ position of the inositol ring [145]. PI3K is induced under physiological conditions by various stimuli, including growth factors, cytokines and hormones that bind to the receptor tyrosine kinase (RTK) supplied with a ligand binding domain. Apart from the extracellular domain, RTK also consists of the transmembrane and intracellular tyrosine kinase domains. The presence of ligand results from two RTKs monomers forming a dimer followed by induction of tyrosine kinase domain in the cytoplasmic region and autophosphorylation of tyrosine residue. Thus, PI3K is recruited to the cell membrane and activated [146]. The class I PI3K that activates AKT is a heterodimer composed of one regulatory and one catalytic subunit. p85α, p55α, p50α, p85β and p55γ are homologous regulatory subunits, whereas p110α, p110β and p110δ are catalytic ones [147]. Phosphatase and tensin homolog (PTEN) and Src homology 2 (SH2) domain containing inositol polyphosphate 5-phosphatase 1 (SHIP1) are specific PI3K inhibitors that act via dephosphorylation of PIP3 into PI(4,5)P2 or PI(3,4)P2, respectively [148,149]. 

A key molecule in PI3K signaling pathway is serine/threonine-specific protein kinase AKT (also named protein kinase B, PKB) with numerous different downstream target proteins. The family includes three isoforms: AKT1, AKT2 and AKT3. AKT is composed of three specific domains: the plekstrin homology (PH), catalytic with ATP-binding sites and regulatory. PI(3,4,5)P3 binds to the PH domain and mediates AKT translocation to the plasma membrane. The activation of AKT is then a result of double phosphorylation in Thr308 and Ser473 sites by phosphoinositide-dependent kinase-1 (PDK-1) localized in membrane and by mammalian target of rapamycin complex 2 (mTORC2), respectively [150]. AKT downstream substrates control among others cell survival, growth and proliferation [151,152]. 

AKT promotes cell survival by phosphorylation of substrates that take part directly in apoptosis pathway, such as proapoptotic proteins, including the Bcl-2 associated agonist of cell death (Bad), and caspase-9 or indirectly, such as Forkhead box O (FOXO), glycogen synthase kinase 3 (GSK3) and murine double minute 2 (MDM2; HDM2 in humans) [151]. Transcription factor FOXO promotes apoptosis in mitochondria-dependent and mitochondria-independent manners via induction of proapoptotic Bcl-2-like protein 11 (Bim) and cluster of differentiation 95 (CD95, Fas) ligand specific for death receptors, respectively [153,154]. GSK3 phosphorylate proapoptotic Bcl-2-associated X protein (Bax). That action mediates Bax mitochondrial translocation, followed by activation and triggering the programmed cell death signaling [155]. AKT-dependent phosphorylation of MDM2 leads to translocation into the nucleus and act as a suppressor of p53. An important prominent outcome of that transcription factor inhibition is the blockage of apoptosis [156]. 

AKT promotes cell growth by activation of mTORC1, which is involved in initiation of translation and the production of ribosomes. AKT acts as a negative regulator of tuberous sclerosis complex 2 (TSC2), a part of the TSC1/TSC2 complex. Complexed TSC2 plays a role as GTPase-activating protein (GAP) for the Ras-related small G protein (Rheb). Switching Rheb to active form deactivates mTORC1. TSC1/2 inhibition through AKT leads to mTORC1 activation via Rheb suppression [157]. Another substrate phosphorylated by AKT is a proline-rich AKT substrate of 40 kDa (PRAS40). PRAS40 is also a component of mTORC1 and a negative regulator of that signaling [158]. Therefore, AKT stimulates mTORC1 activation via repression of its suppressors such as TSC2 and PRAS40 [151]. Activated mTORC1 inhibits autophagy induction by direct phosphorylation of unc-51-like kinase 1 (ULK1) and ULK2 (ULK complex). ULK is responsible for autophagy induction [159]. 

AKT promotes cell proliferation by downstream target proteins that regulate cell cycle. AKT phosphorylates p27 and p21, the negative regulators of cyclin-dependent kinase (CDK) 2 and CDK4/6. CDK2 complexed with cyclin A/E and CDK4/6 complexed with cyclin D phosphorylate the retinoblastoma protein (pRB) followed by their inactivation. As a consequence of this action, E2F transcription factor is released, thus inducing several genes that promote cell growth and enter cell into the synthesis (S) phase [160]. Moreover, phosphorylation of targets, including GSK3, TSC2 and PRAS40, is also related to cell cycle modulation. GSK3 regulates the S phase transition by influencing cyclin D expression via impact on β-catenin, a component of Wnt pathway. In the absence of Wnt ligands, a multiprotein complex that contains GSK3 as well as axin, adenomatosis polyposis coli (APC), protein phosphatase 2A (PP2A), casein kinase 1α (CK1) and β-catenin are located in the cytosol. β-catenin is phosphorylated by both GSK3 and CK1, followed by their proteasomal degradation. In the presence of Wnt ligands, it binds to a receptor called Frizzled (Fz) and low-density-lipoprotein-related protein 5/6 (LRP5/6), a receptor-related protein. Then, APC/axin/GSK3 complex translocates into the membrane and binds to the cytoplasmic domain of LRP5/6. The binding of axin to the receptor-related protein is enabled by phosphorylation that mediates CK1 or GSK3. Thus, the role of GSK3 is multifaced: positive and negative via LRP5/6 and β-catenin phosphorylation, respectively. Moreover, scaffold protein Dishevelled (Dsh) is recruited to Fz. Dsh is phosphorylated by several kinases including CK1. Dsh acts as a negative regulator of GSK3 enzyme and disables GSK3-dependent phosphorylation of β-catenin followed by prevention of their degradation. Signaling events lead to β-catenin stabilization and their translocation into the nucleus, which induces the target genes [161,162], including cyclin D, consequently resulting in progression of the cell cycle [163]. Inhibition of GSK3 via AKT induces a longer mitotic arrest in the presence of taxol compared to taxol alone [164]. Moreover, phosphorylation of TSC2 and PRAS40 activate mTORC1 pathway, also implicated in the cell cycle progression. mTORC 1 regulate cyclin levels by influencing their synthesis and/or degradation as well as playing a role as a repressor of p21 and p27 [165].

### 5.2. PI3K/AKT Pathway Regulate Macrophage Polarization

The PI3K/AKT cascade contributes to macrophage polarization. PI3K and AKT isoform-specific effects have been reported [166]. The study concerning the usage of PI3K/AKT inhibitor named LY294002 confirmed that PI3K/AKT signaling is related to M2 polarization in bone-marrow-derived macrophages from emphysematous mice and cigarette-smoke-extract-treated RAW264.7 cells [167]. Another work based on LPS-stimulated RAW 264.7 treated with two inhibitors of PI3K, named wortmannin and LY294002, show elevated levels of iNOS. Moreover, incubation with wortmannin mediates activation of NF-κB [168]. The following study describes a dual inhibitor, named SRX3207, of spleen tyrosine kinase (Syk) and a downstream phosphorylated effector PI3K. The data show that blockage of Syk/PI3K axis promotes a proinflammatory M1 phenotype in macrophages derived from the bone marrow of mice [169]. Separate data indicate that PI3Kγ knockout mice peritoneal macrophages induce NF-κB activation and IL-1β release, characteristic for M1 phenotype [170]. In response to stimulation with LPS and IFN-γ, macrophages abrogated with PI3K p85α regulatory subunit exhibit improper production of NO and IL-12, which are essential compounds to defeat pathogen invasion [171]. The study indicates that TLR signals lead to activation of PI3K [11], but that kinase is a suppressor of IL-12 release by TLR induction, thus blocking excessive innate immune response [172].

Deletion of AKT1 promotes M1 macrophages, whereas ablation of AKT2 gives rise to the M2 macrophage phenotype [14]. Inhibitors of AKT1 play a role in prevention of intracellular growth of various bacteria [173], whereas AKT2 deletion prevents macrophages from undergoing M1 polarization, reduces expression of proinflammatory genes and limits cell migration [174]. In addition, AKT regulates the response of macrophages to LPS via control of miRNA expression. AKT1 ablation in RAW264.7 macrophages exhibit elevated responsiveness to LPS whereas excessive expression of let-7e and blocked expression of miR-155 in AKT1 knockout macrophages restores proper sensitivity to LPS [175]. Another study shows that AKT2 ablation in LPS-activated RAW264.7 cells exhibit suppressed expression of miR-155 and upregulated level of CCAAT/enhancer-binding protein β (C/EBPβ) transcription factors, important agents implicated into M2 phenotype regulation [14]. Additionally, deletion of PDPK1, which plays an important role in AKT activation, enhances M1 macrophages polarization in mice adipose tissue macrophages [176]. The data suggest that AKT1/AKT2 balance influences mTORC1 activity and plays a crucial role in differentiation of PI3K/AKT-mediated macrophages [177]. 

### 5.3. The Role of the Upstream Regulators of AKT in Macrophages Polarization—PTEN, SHIP, PDK-1

Loss of PTEN results in increased AKT activity inhibited responses to LPS and M2 polarization [178]. In addition, PTEN loss in mice peritoneal macrophages show increased expression of C/EBPβ and STAT3 [179]. The expression of M2 markers was also higher, whereas production of TNF-α and IL-10 in response to TLR ligands was lower and higher in PTEN knockout mice peritoneal macrophages, respectively [180]. Furthermore, LPS induction of TNF-α, IL-6 was decreased in PTEN-abrogated mice peritoneal macrophages [178].

PI3K/AKT activity is related to a loss of SHIP and M2 phenotype [13]. Additionally, reduced SHIP levels lead to enhanced STAT6 transcription in macrophages derived from the bone marrow of mice [181]. SHIP inhibits the M2 macrophages differentiation via suppression of IL-4 release from basophils [182]. Thus, M2 macrophages activation by IL-4 requires SHIP degradation [13]. 

Ablation of PDK-1 predisposes to the M1 phenotype and attenuates an inflammatory response via limitation of aerobic glycolysis in these cells. Furthermore, enhanced PDK-1 leads to the M2 phenotype by increased mitochondrial respiration needed in the early step of activation of these cells [183]. 

### 5.4. The Role of AKT Downstream Signal in Macrophage Polarization—mTOR

The TSC/mTORC 1 pathway is a downstream effector of PI3K/AKT signaling and a crucial sensor of nutrient status. That pathway also plays an important role in coordination of inflammatory stimuli and determines the macrophage activation phenotype. mTORC1 activation is mediated by AKT [12]. The data indicate that TSC1 and TSC2 play a crucial role in macrophage polarization. TSC1-abrogated peritoneal mice macrophages exhibit elevation of the M1 and M2 phenotypes in both steady-state and inflammatory conditions. In addition, those macrophages exhibit impaired migration and reduced expression of chemokine receptors such as CCR2 and CCR5 [184]. Another study indicates that inhibition of mTOR1 by rapamycin is followed by a release of proinflammatory agents by the NF-kB pathway but inhibits the release of interleukin-10 by STAT3, whereas the trends change in TSC2 knockout human peripheral blood mononuclear cells, and NF-kB and STAT3 activity were reduced and enhanced, respectively, in relation to M2 differentiation [185]. Inhibition of mTORC1 via rapamycin promotes M1 macrophage polarization in human macrophages [15]. Thus, induction of the PI3K/AKT/mTOR1 signaling cascade promotes M2 macrophage polarization. It has been proposed that the AKT/mTORC1 axis shifts metabolism to the high-energy supply demand of M2 differentiation [186]. 

### 5.5. The Contribution of AKT Signaling in Phagocytosis and Autophagy

Elimination of pathogens by phagocytosis is an important attribute of macrophages that initiate innate immune response, the first line of defense. The innate immune response contributes to the activation of adaptive immunity [187,188]. Macrophages, in response to chemotactic signals, are recruited to the inflammation site. That cells have a restricted number of phagocytic receptors, including mannose receptors. Mannose receptors act as pathogen conserved motif recognition receptors. In addition, complement receptors and Fc receptors take part in phagocytosis of nonspecific or specific with antibodies opsonized pathogens, respectively [187]. The clearance of foreign particles is induced by rearrangement in the cell cytoskeleton, which leads to their internalization into vesicles [189]. Actin polymerizations promote formation of filopodia, the structure that enables movement to the inflammation site. The study investigated gene silencing against PI3K and mTOR in macrophages indicates that PI3K ablation group exhibited short disorganized filopodia, whereas mTOR ablation group show long, dense ones [190]. AKT2 is also essential for tumor-associated macrophages chemotaxis [191]. 

Pathogens are eliminated in the autophagy process by the formation of double-membrane vesicles named autophagosomes which are fused with lysosomes. Autophagy is regulated by mTOR pathway [192]. The research has demonstrated that inhibition of autophagy in tumor-associated macrophages may attenuate M2 macrophage polarization [193]. In addition, activation of mTOR in bone-marrow-derived macrophages can increase M2 polarization [194]. Furthermore, human peripheral monocytes stimulated by LPS and treated with rapamycin, an mTOR inhibitor, exhibit M1 phenotype, while TCS2 knockdown provides differentiation into M2 macrophages [195].

### 5.6. The Contribution of Macrophage AKT in Inflammatory Diseases

Inflammation is an important mechanism implicated in both human health and disease. Physiologically, that sequence of reactions removes invaders and promotes repair of damaged tissue [196]. Macrophages participate in every step of the inflammation process, including initiation, maintenance and resolution, and play a role in antigen presentation, phagocytosis and immunomodulation. Additionally, macrophages mediate restoration of tissue function and homeostasis [197,198]. Overall, macrophages act as positive as well as negative regulators of inflammatory response. However, chronic inflammation can lead to tissue injury and diseases. Related disorders are as follows: diabetes, cardiovascular diseases, chronic respiratory diseases, arthritis, cancer and obesity. Long-term inflammation is an important cause of death worldwide [199].

The study conducted on mouse model of endotoxemia indicated that the PI3K/AKT pathway inhibit LPS-induced inflammation [200]. In another study, it was reported that AKT2 knockout mice were more resistant to LPS-induced inflammation as well as dextransulfate-sodium-induced colitis [14]. The next observation suggests that AKT1 knockout mice are more sensitive to pulmonary fibrosis development [201]. 

## 6. Plants as Natural Modulators of PI3K/AKT Pathway in Macrophages

The PI3K/AKT/mTORC1 pathway is widely recognized as a signaling pathway involved in normal cell physiology. It has been shown to be capable of inhibiting apoptosis and promoting the cell cycle progression while also inhibiting autophagy [202]. Autophagy is a potent anti-inflammatory process that influences the physiology of inflammatory cells, including macrophages. In addition, that process mediates the release of inflammatory and antimicrobial mediators, inhibits inflammasome activation and enhances phagosome maturation [16,203]. Inhibition of the PI3K/AKT/mTORC1 axis results in autophagy activation and protection of macrophages cells from inflammation. That signaling could be suppressed by selected plant extracts as well as pure compounds with anti-inflammatory properties.

This section is focused on a detailed summary of the research progress on the plant extracts as well as single derived compounds with anti-inflammatory potency in macrophages, especially via inhibition of the PI3K/AKT/mTOR axis. The mechanisms of action, macrophage cell lines and specific types of extracts derived from different parts of plants that belonged to various families are presented in Table 1, whereas single derived compounds are presented in Table 2. The list includes publications from the last five years. 

## 7. Conclusions

The plant kingdom is a source of important bioactive compounds with a wide range of applications in medicine. Hence, this review highlights the efficient anti-inflammatory activities of extracts or pure chemicals via modulating various signaling pathways, especially PI3K/AKT in macrophage cell cultures. Macrophages are positive as well as negative regulators of inflammatory response. Regulators of their biology are important immune targets. However, further in vivo studies are necessary to fully explore bioavailability and biofunctionality of plant-derived compounds and the molecular mechanisms of action under physiological conditions. Despite the continued presence of many questions in that field, the value of secondary metabolites is important for the development promising molecules. Regarded as safe, phytochemicals may constitute novel therapeutic approaches against inflammatory disorders.

## Figures and Tables

**Table 1:** Selected plant extracts with potential regulatory activity on macrophages’ biology related to modulation of the PI3K/AKT pathway.

Name of the Species	Name of the Family	Part of the Plant	Type of Extract	Determined Chemicals	Cell Line	Mechanism of Action	Ref.
*Apios americana* Medikus	Fabaceae	leaves	ethanolic	vicenin-2, schaftoside, baimaside, apigenin (6-*C*-*α*-l-arabinopyranosyl)-8-*C*-*β*-d-glucopyranoside, vitexin, isquercetin, 6-*C*-*β*-d-glucopyranosyl-7-*O*-methylluteolin	RAW 264.7 cells	Inhibition of AKT and mTORC1 activity	[102]
*Artocarpus lakoocha* Roxb.	Moraceae	heartwood	ethanolic	oxyresveratrol (trans-2,30,4,50-tetrahydroxystilbene)	RAW 264.7 cells	Inhibition of AKT activity	[204]
*Aster incisus* Fisch.	Asteraceae	whole plant	methanolic	4-((1*E*)-3-Hydroxy-1-propenyl)-2-methoxyphenol, liliolide, neophytadiene, triterpene lupeol, trans-phytol, palmitic acid beta-monoglyceride, chondrillasterol, olean-12-en-3-one, palmitic acid	RAW 264.7 cells	Inhibition of PI3K and AKT proteins expression	[109]
*Aster yomena* (Kitam.) Honda	Asteraceae	leaves	ethanolic	-	RAW 264.7 cells	Inhibition of PI3K and AKT activity	[205]
*Astragalus membranaceus* (Fisch.) Bunge	Fabaceae	whole plant	-	-	ANA-1 cells	Inhibition of AKT activity	[206]
*Chrysanthemum indicum* L.	Asteraceae		methanolic	luteolin	RAW264.7 cells	Inhibition of AKT1 and AKT2 activity	[207]
*Dimocarpus longan* Lour.	Sapindaceae	flowers, seeds, and pulp	aqueous	corilagin, ellagic acid, and gallic acid	RAW 264.7 cells	Inhibition of AKT activity	[208]
*Eleutherococcus senticosus* (Rupr. et Maxim.) Maxim	Araliaceae	roots and bark	root—ethanolic bark—methanolic	root extract containing more syringin, caffeic acid, and isofraxidin than the bark the bark extract contained more sesamin and oleanolic acid	human primary macrophages	Inhibition of AKT activity	[209]
*Eucommia ulmoides* Oliv.	Eucommiaceae	bark	aqueous	pinoresinol diglucoside, geniposide, aucubin	RAW 264.7 cells	Inhibition of PI3K, AKT, mTORC1 activity	[210]
*Gouania leptostachya* DC. var. tonkinensis Pitard.	Rhamnaceae	leaves	methanolic	resveratrol	RAW264.7 cells and mouse peritoneal macrophages	Inhibition of AKT activity	[211]
*Mahonia bealei* (Fort.) Carr.	Berberidaceae	leaves	aqueous extract and fractionated using a series of organic solvents, including n-hexane, dichloromethane, ethylacetate, n-butanol	-	RAW 264.7 cells	Inhibition of PI3K, AKT activity	[212]
*Mycetia cauliflora* Reinw.	Rubiaceae	aerial parts	methanolic	-	RAW 264.7 cells	Inhibition of AKT activity	[213]
*Panax ginseng* C.A. Meyer	Araliaceae	roots	aqueous	-	RAW 264.7 cells	Activation of PI3K and AKT activity	[214]
*Panax ginseng* Mayer	Araliaceae	berry calyx	ethanolic	ginsenosides	RAW264.7 cells	Inhibition of AKT1 and AKT2 activity	[215]
*Panax ginseng* Meyer	Araliaceae	roots	aqueous	ginsenosides	RAW264.7 cells	Induction of PI3K, AKT activity	[216]
*Persicaria chinensis* L.	Polygonaceae	aerial parts	methanolic	caffeic acid, kaempferol, and quercetin	peritoneal macrophages, and RAW264.7 cells	Inhibition of AKT activity	[217]
*Phyllanthus acidus* (L.) Skeels	Phyllanthaceae	leaves	methanolic	quercetin, kaempferol, caffeic acid	RAW 264.7 cells, U937 cells	Inhibition of AKT activity	[218]
*Phyllanthus amarus* Schum. & Thonn.	Phyllanthaceae	whole plant	ethanolic	lignans, phyllanthin, hypophyllahtin and niranthin, and polyphenols, gallic acid, geraniin, corilagin, ellagic acid	U937 cells	Inhibition of AKT protein expression	[104]
*Piper cubeba* L.	Piperaceae	whole plant	methanolic	quercetin, luteolin, and kaempferol	RAW 264.7 cells	Inhibition of PI3K and AKT activity	[66]
*Saussurea involucrate* (Kar. et Kir.) Sch.-Bip.	Asteraceae	aerial parts	methanolic	rutin, chlorogenic acid	RAW 264.7 cells	Inhibition of PI3K and AKT activity	[219]
*Toona sinensis* (Juss.) M. Roem	Meliaceae	leaves	aqueous	gallic acid, methyl gallate, ethyl gallate, kaempferol, kaempferol 3-*O*-b-d-glucoside, quercetin, quercitrin, quercetin 3-*O*-b-d-glucoside, rutin	RAW 264.7 cells	Inhibition of mTORC1 activity	[220]
*Torilis japonica* (Houtt.) D.C.	Umbelliferae	fructus	ethanolic	-	RAW 264.7 cells	Inhibition of AKT activity	[221]
*Trigonostemon reidioides* (Kurz). Craib	Euphorbiaceae	stems	ethanolic	-	RAW 264.7 cells	Inhibition of PI3K, AKT activity	[222]
*Zingiber zerumbet* (L.) Roscoe ex Sm.	Zingiberaceae	rhizomes	ethanolic	zerumbone, ethyl gallate, gallic acid, catechin, kaempferol rhamnoside, kaempferol, kaempferol-3-*O*-(2”, 4”-diacetyl) rhamnoside isomers, kaempferol methylether, kaempferol methylether isomer, kaempferol-3-*O*-(3”,4”-diacetyl) rhamnoside isomers, kaempferol glucoside conjugate, demethoxycurcumin, curcumin, bisdemethoxycurcumin	U937 cells	Inhibition of AKT activity	[101]

**Table 2:** Plant single derived compounds with potential regulatory activity on macrophages’ biology related to modulation of the PI3K/AKT pathway.

Name of the Species	Name of the Family	Part of the Plant	Name of the Chemical	Class of the Chemical	Cell Line	Mechanism of Action	Ref.
*Ailanthus altissima* (Mill.) Swingle	Simarubaceae	stem bark	canthin-6-one	alkaloid	RAW 264.7 cells	Inhibition of AKT activity	[223]
*Apios americana* Medikus	Fabaceae	tubers	polysaccharide (glucose, arabinose, galactose and galacturonic acid)	polysaccharide	RAW 264.7 cells	Inhibition of AKT and mTOR activity	[224]
*Bletilla striata* (Thunb.) Rchb.f.	Orchidaceae	whole plant	coelonin	dihydrophenanthrene	RAW 264.7 cells	Inhibition of PI3K and AKT activity	[225]
*Cirsium japonicum* Fisch. ex DC.	Asteraceae	aerial parts	cirsimaritin	flavonoid	RAW 264.7 cells	Inhibition of AKT activity	[226]
*Etlingera pavieana* (Pierre ex Gagnep) R.M.Sm.	Zingiberaceae	rhizomes	4-methoxycinnamyl p-coumarate	phenolic compound	RAW 264.7 cells	Inhibition of AKT activity	[227]
*Mahonia bealei* (Fortune) Carrière	Berberidaceae	leaves	20-hydroxy-3-oxolupan-28-oic acid	triterpene	RAW 264.7 cells	Inhibition of PI3K and AKT activity	[228]
*Paeonia lactiflora* Pall.	Paeoniaceae	rhizomes	4-*O*-methylbenzoylpaeoniflorin	monoterpene	RAW 264.7 cells	Inhibition of PI3K and AKT activity	[229]
*Physalis peruviana* L.	Solanaceae	aerial part	4β-hydroxywithanolide E and physalactone	withanolides	RAW 264.7 cells	Inhibition of AKT activity	[230]
*Solanum nigrum* L.	Solanaceae	fruits	solanine A	alkaloid	RAW 264.7 cells	Inhibition of AKT activity	[231]
*Sophora alopecuroides* L.	Fabaceae	roots	sophoraflavanone G	flavonoid	RAW 264.7 cells	Inhibition of PI3K and AKT activity	[232]
*Swertia chirayita* (Roxb. ex Fleming) H. Karst.	Gentianaceae	whole plant	bellidifolin	xanthone	RAW 264.7 cells	Inhibition of AKT activity	[118]
*Trigonostemon reidioides* (Kurz) Craib.	Euphorbiaceae	roots	trigonoreidon B	diterpene	RAW 264.7 cells	Inhibition of PI3K and AKT activity	[233]
*Trillium tschonoskii* Maxim.	Liliaceae	roots and rhizomes	urotrilliumoside	saponin	RAW 264.7 cells	Inhibition of PI3K and AKT activity	[234]
*Zingiber zerumbet* (L.) Sm.	Zingiberaceae	rhizomes	zerumbone	sesquiterpene	U937 cells	Inhibition of AKT activity	[235]
